# Supplementary material for: Determinants of anxiety levels among young males in a threat of experiencing military conflict–Applying a machine-learning algorithm in a psychosociological study
Source: PLoS One. 2020 Oct 7;15(10):e0239749. doi: 10.1371/journal.pone.0239749 (PMC7540846; doi:10.1371/journal.pone.0239749)
Supplement: Attachment — Submitted filename: response to editor.docx [file pone.0239749.s001.docx]

**Response to Editor Comments:**

*1. Title page (Methods) – Does not mention any data collected that was relevant to military action?*

Action: Description added

**Methods**

A sample of Ukrainian males, in duty as conscripts to military service (n=392, M±SD=22.1±5.3) participated in a survey. We used the 36-Item Short Form Health Survey, and State-Trait Anxiety Inventory. In addition to psychological indices, social-demographic data were collected. To discover the number of clusters, the k-means algorithm was used, the optimal number of clusters was found by the elbow algorithm. For validation of the model and its use for further prediction, the random forest machine-learning algorithm, was used.

*2. Page 9 Not really relevant – and PTSD is no longer defined by traumatic physical injury*

Action: Explanation added

Currently, PTSD is no longer associate with the direct physical stressor, but also is believed to be caused by indirect exposure to aversive details of the trauma or learning that a relative or close friend was exposed to a trauma (5).

*3. Page 10 This belongs in the methods, not background*

Action: Part transferred to methods.

*4. Page 11 – ???*

Action – deleted

*5. Page 11 Need to provide an overview of this for naive readers*

Action: Overview added

The conflict started with the increased insurgency against the newly established government after the removal of Yanukovych government in the south-east region of Ukraine. Then armed conflict started as Russian soldiers without any military insignia manipulated those areas and declare separation from the rest of the country and finally annexation of Crimea, which is not recognized by international institutions. Since then, there was an anti-terrorist operation ongoing, which is officially held by the Ukrainian military since 2018, when president Poroshenko announced that aggression is caused by the Russian Federation. Conflict status is ongoing. Ukraine has conscript army, so the students of different military-related studies will have to be on duty at some point.

*6. Page 12 this is methods, not background*

Action: Transferred

*7. Page 13 Participants, not "data selection. This needs to describe how participants were recruited/invited into the study.*

Action: Extended description provided.

A sample of Ukrainian males (n=392, M±SD=22.1±5.3) participated in a survey.

Four groups of respondents were involved in the study:

- students of military specialization,
- internal affairs specialization,
- sport specializations,
- military service personnel.

Universities offers reserve officer training for two years. An alternative to that is one-year regular service as conscript troops. Maximum conscription age is 27 years.

Personal characteristics of participants included gender, age in years, education level (secondary education, bachelor’s degree, master’s degree, Ph.D. degree), marital status (never married, married, divorced/live separate, widow/widower, civil marriage), length of service (in months), residence time in conflict zone, the existence of war injuries, and duration of rehabilitation (in months) if person sustained injury in the military zone. Sociodemographic data reflected participants' status as of September, 2018.

All participants agreed to engage in the study and provided written informed consent. The participants were informed about the purpose of the study and were informed that their participation would be voluntary and that they could withdraw from the study at any moment. The participants were also apprised that the collected information would be anonymous, and individual data could not be identified.

After giving written informed consent, each participant was asked to complete the self-administered questionnaire in hard copy.

*8. Page 14 Separate this out into an another section, and describe the SF-36 on its own.*

Action: Separated

*9. Page 14 –*

all concerns about protocol, data acquisition and collection was extended.

*10. Page 15 – What does this mean? Rows usually pertain to the individual participant, and the variables are in columns.*

Action: Rows changed to participants

*11. Page 15 - What were the binary classifications? And Page 16 Again, what was the cut point for the binary variable?*

Action: Description of binarization process was provided with suitable reference.

*12. Page 16 – Please explain how this was used to identify the number of clusters?*

Action: The optimal number of clusters was found by the elbow algorithm, which is based on the sum of the squared distances between data points and their assigned cluster centroids. This method is used to identify the maximal number of efficient clusters. Adding more clusters than obtained from an elbow algorithm does not contribute to better modelling of data.

*13. Page 17 – Clarify explicitly whether/how these data were used for validation?*

Data was split into groups for training the model purposes (80%) and some portion of the data (20%) was kept aside for a purpose of testing obtained models. After training the model, these test data was used for validation

*14. Page 17 – I'm not sure that this is the correct word. If it is correct, please explain where they were enrolled as students?*

Action: Explanation added in methods.

Universities offers reserve officer training for two years. An alternative to that is one-year regular service as conscript troops. Maximum conscription age is 27 years.

*15. Page 17 – There is no such thing as "highly" nonsignificant.*

*Please define the criteria used to determine statistical significance in the data analysis section of the methods.*

Action: Overall level of significance was used as common practice on p < 0.05.

*16. Page 18 – If it was not significant then you cannot describe it as if it is an actual difference between the groups.*

Action: Deleted.

*17. Page 18 This belongs in the data analysis section in the methods. In this section you report the results with those meanings in mind.*

Action: Moved.

*18. Page 18 – Be specific here that you're talking about the number of people in each cluster varying from 45-120.*

Action: Description added:

By performing k-means cluster analyses, 3 clusters (subgroups) were identified, with cluster sizes ranging from 45 to 120 participants (Figure 3).

*19. Page 18 – The methods indicated that the cluster analyses used the STAI and SF-36 variables, so you need to describe how the clusters were defined in relation to THOSE variables/characteristics before going on to differentiate the clusters based on the other demographic characteristics.*

*Give the clusters a descriptive name in the body of the results and in the tables so that your explanation of their demographic characteristics can be considered in light of those anxiety/health-related characteristics.*

Action: Names was changed to subgroups and this section was reworked

*20. Page 20 – This is not a very clear explanation of the findings.*

Action: Previous action was connected with this comment as well.

*21. Page 20 – It is better describe what the analysis DOES allow you to understand rather than what id DOES NOT help you to understand about the models. Or should this sentence not include the term "not"?*

Action: Commented sentence is conclusion about not detecting strict and easy pattern regarding our assumption. Not correction provided.

*22. Page 21 – Should this be a part of the previous paragraph?*

Action: Corrected

*23. Page 21 – This is a very broad and vague sentence about health in general, but the discussion should contextualise the findings in terms of the study aims.*

Action: Sentences changed.

In the presented study, we outline hat type of perception of quality of life, which plays a significant role in the perception of good well-being, perceived emotion and daily life function (13) in the context of living in a threat of possible engagement in military actions. The applied model of social science computational techniques allowed us to successfully classify participants by their biopsychosocial profiles for 3 subgroups (clusters).

*24. Page 22 – This is quite a technical description of the methods that sounds like you're highlighting limitations of the study. I suggest you go straight to summarising the findings, and THEN if relevant mentioning the technical qualifiers/limitations.*

Action: Paragraph removed.

*25. Page 22 – Need to link everything in the discussion to the methods/results of this study, otherwise broad statements on the topic or of little relevance.*

*Structure your paragraphs so that they mention/highlight key findings and then contextualise to existing literature/theory etc.*

Action: Paragraphs were reorganised.

*26. Page 23 – Do not refer to tables/figures of results in the discussion. You are only discussing and the interpreting findings in the discussion.*

*Also, here is the first time you're mentioning "stens" scale. It is not clear to the reader what you mean.*

Action: Stens explanation provided in methods. References to tables deteled.

*27. Page 26 – What tested groups?*

Action: Sentence deleted, as reorganisation of section omit this concern now.

*28. Page 26 – It is important to include a discussion of the study limitations before the conclusions section.*

Limitation of study is described in comment 21.

*29. Page 27 – Present the conclusions in a cohesive paragraph, rather than a list of sentences.*

*The conclusions do not all flow clearly from the research findings.*

Action: Conclusion section was reorganised.

30. Tables and figures were better described, and tables were transformed accordingly to the requested criteria.
